# The role of copper dysregulation in Wilson disease: an expert opinion

**DOI:** 10.3389/fmed.2025.1673283

**Published:** 2025-10-20

**Authors:** Wolfgang Stremmel, Ralf Weiskirchen

**Affiliations:** ^1^Medical Center Baden-Baden, Internal Medicine, Baden-Baden, Germany; ^2^Institute of Molecular Pathobiochemistry, Experimental Gene Therapy and Clinical Chemistry (IFMPEGKC), RWTH University Hospital Aachen, Aachen, Germany

**Keywords:** liver, brain, ATP7B, mutation, copper, Wilson disease

## Abstract

The handling of free copper is a crucial aspect of copper metabolism. Any dysfunction in this process can lead to the pathophysiology of diseases, such as Wilson disease. This disorder, characterized by an excess of copper in the liver, occurs when the body is unable to excrete copper into bile. The symptoms of the disease result from the accumulation of free copper in liver cells, leading to hepatocellular injury and the release of copper into the bloodstream. This, in turn, causes damage in other areas of the body, such as the brain. The primary goal of therapy is to convert toxic free copper into harmless complexes, rather than simply removing copper from the body.

## 1 Introduction

The recommended daily allowance for copper consumption is 900 µg, but the requirement varies depending on age (1). In the diet, copper is mostly found in the harmless cupric form (Cu^2+^). The cuprous form (Cu^+^), however, is more aggressive as it can produce free radicals within cells. To neutralize this, it must bind to specific proteins with an affinity for copper. Only Cu^+^ can pass through plasma membranes to enter cells and distribute into cell organelles. There is a constant switch between Cu^+^ and Cu^2+^. Copper is classified as a trace metal, with about 50% of copper being absorbed. Biliary secreted copper forms a complex with bile acids that is not absorbable (2). Copper is essential for electron transfer reactions in cellular respiration, including the handling of oxygen and free radicals (3). Copper induced oxidative stress and cuproptosis are the toxic sides of copper overload (4).

## 2 Methodology

Starting in 1981 with the first publication on Wilson disease (WD), we have published over 100 papers on this topic covering clinical and laboratory aspects. Our experience with WD is based on the care of approximately 150 affected patients over 23 years. It began with Professor J. Lange (Bonn/Gummersbach), a pioneer of WD in Germany in the 1950s to 1970s, who, after retiring, transferred his patients to Professor G. Strohmeyer and Dr. W. Stremmel in Düsseldorf. The responsibility for these patients included the scientific evaluation of the disease and participation in international conferences on WD. Personal contacts with the founders of current views on WD significantly expanded our knowledge, including Drs. J. P. Walsh, I. H. Scheinberg, I. Sternlieb, G. J. Brewer, T. U. Hoogenraad, among others.

The present manuscript originated from an oral presentation at the webinar “*Metabolic Mysteries: The Role of Copper Dysregulation in Liver Disease*”.^1^ We included essential practical aspects in the diagnosis and management of WD without claiming to be comprehensive. We referred to current guidelines that are extensive and up to date (5, 6). However, some aspects reported in guidelines are not practical in the daily routine of family physicians but are rather theoretical considerations based on new scientific results. The aim of the presentation was to elucidate the underlying pathophysiology of WD to understand the usefulness of current diagnostic and therapeutic approaches. Based on the hypothesis that free copper leads to disease manifestation, we selected appropriate publications addressing this topic over the last 50 years. The quality of the scientific data was considered more important than the date of publication. The conclusions drawn by us should be considered subjective. We do not claim to know the truth. Therefore, the paper should to be considered an expert opinion.

## 3 Physiology of copper absorption

**Figure1:**
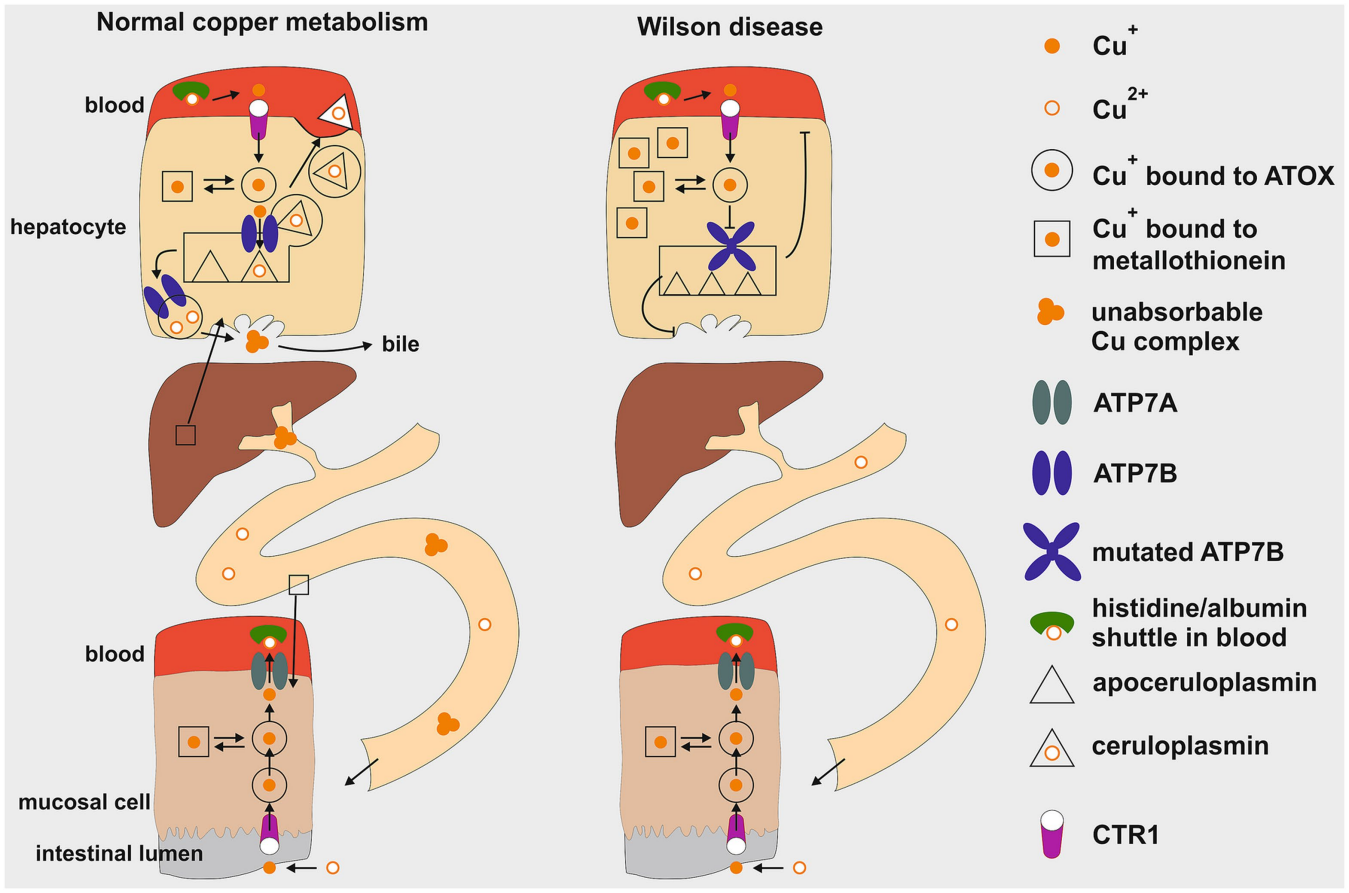
Schematic illustration of copper metabolism in healthy individuals compared to Wilson disease. The regulation of copper levels involves absorption in the upper gastrointestinal tract and excretion by liver cells (hepatocytes) into bile as an insoluble complex. Key components include the mucosal cell and the hepatocyte. This diagram illustrates the processes of cellular uptake, intracellular transport, storage, metabolic processing within the liver, and cellular excretion. In Wilson disease a mutation in the ATP7B gene leads to a defective copper transporter at the trans-Golgi network, hindering proper loading of apoceruloplasmin with copper, resulting in decreased serum copper levels. Additionally, impaired biliary excretion causes copper accumulation in hepatocytes, initially bound to metallothionein. Cu^+^, cuprous form; Cu2^+^, cupric form; ATOX1, antioxidant copper chaperone; ATP7A, ATPase copper transporter alpha; ATP7B, ATPase copper transporter beta; CTR1, copper transporter 1. This figure was taken from Stremmel and Weiskirchen (9).

As illustrated in Figure 1, the uptake of copper into cells occurs only in the Cu^+^ state and is mediated by the copper transporter 1 (CTR1) (7, 8). It is debated whether a separate, but associated reductase is required or if CTR1 contains reductase activity within itself. The first step is the passage across the intestinal mucosa cell. After CTR1-mediated uptake, copper is bound within the cell to the antioxidant Cu chaperone 1 (ATOX1), which directs Cu^+^ to Cu-transporting P-type ATPases (9–11). In the intestine, the Menkes disease protein ATPase copper transporter alpha (ATP7A) translocates Cu^+^ out of the mucosa cell. Dephosphorylation of the protein at methionine and histidine residues is required for its external release from ATP7A (9, 11). From there, copper in its oxidized Cu^2+^ form is taken up by albumin, to which it is loosely associated, and exchanges with free histidine in the blood. After dissociation of Cu^2+^ from albumin and reduction to Cu^+^, the transport into cells is mediated by CTR1. Only a small fraction of serum copper is bound to the Small Copper Carrier (SCC), a peptide of 2 kDa, which transports copper in the urine (<50 ug /day) (12). Thus, urinary copper release is of marginal physiological significance in healthy individuals. In hepatocytes after cellular uptake via CTR1, Cu^+^ is bound to ATOX1 from where it is transferred to the WD protein ATP7B. Located at the trans-Golgi network membrane, ATP7B transfers Cu^+^ to the Golgi lumen, where it is loaded onto apoceruloplasmin. Human ceruloplasmin contains two types of copper-binding sites: three mononuclear “type 1” sites (one in domains 2, 4, and 6) and a histidine-rich trinuclear cluster site (formed between domains 1 and 6), which functions as the catalytic core. These six integral copper atoms, along with two additional labile sites, are crucial for its function in iron homeostasis and as a ferroxidase enzyme required for cellular iron excretion (13, 14). Thus, ceruloplasmin plays no functional role in copper metabolism. A conventional view proposes that apoceruloplasmin loaded with Cu^2+^ ions is being released as ceruloplasmin by vesicular excretion at the basolateral plasma membrane into the blood to exert its function (15). Non copper-bound ceruloplasmin is less stable and degraded in blood. An alternative view suggests that apoceruloplasmin (non-copper-bound ceruloplasmin) in the blood is of quantitative significance and may have functional activity (16). Nevertheless ceruloplasmin is the most abundant copper-carrying protein in the blood (95%), while albumin contributes only 5% to copper binding proteins.

When the intracellular Cu^+^ load exceeds the capacity of ATOX1 binding, this copper can be stored within metallothionein before being rechanneled for metabolic disposition. Additionally, CTR1 can also operate in the reverse excretion pathway, releasing Cu^+^ back into the blood for distribution via binding to albumin to other organs, such as the brain or kidneys (7, 8).

The mechanism for maintaining a copper balance in the body involves biliary excretion, which begins at the trans-Golgi network, once the need for ceruloplasmin synthesis as well as other copper requiring proteins has been met. Any excess of unused Cu^2+^ is stored in trans-Golgi derived vesicles that are loaded by ATP7B and transported to the canalicular plasma membranes of hepatocytes (17, 18). Upon fusion with these membranes, copper is released into bile and binds to bile acids, forming a nonabsorbable complex that is excreted in stool (2, 11).

## 4 Pathophysiology of copper metabolism in Wilson disease

**Figure 2:**
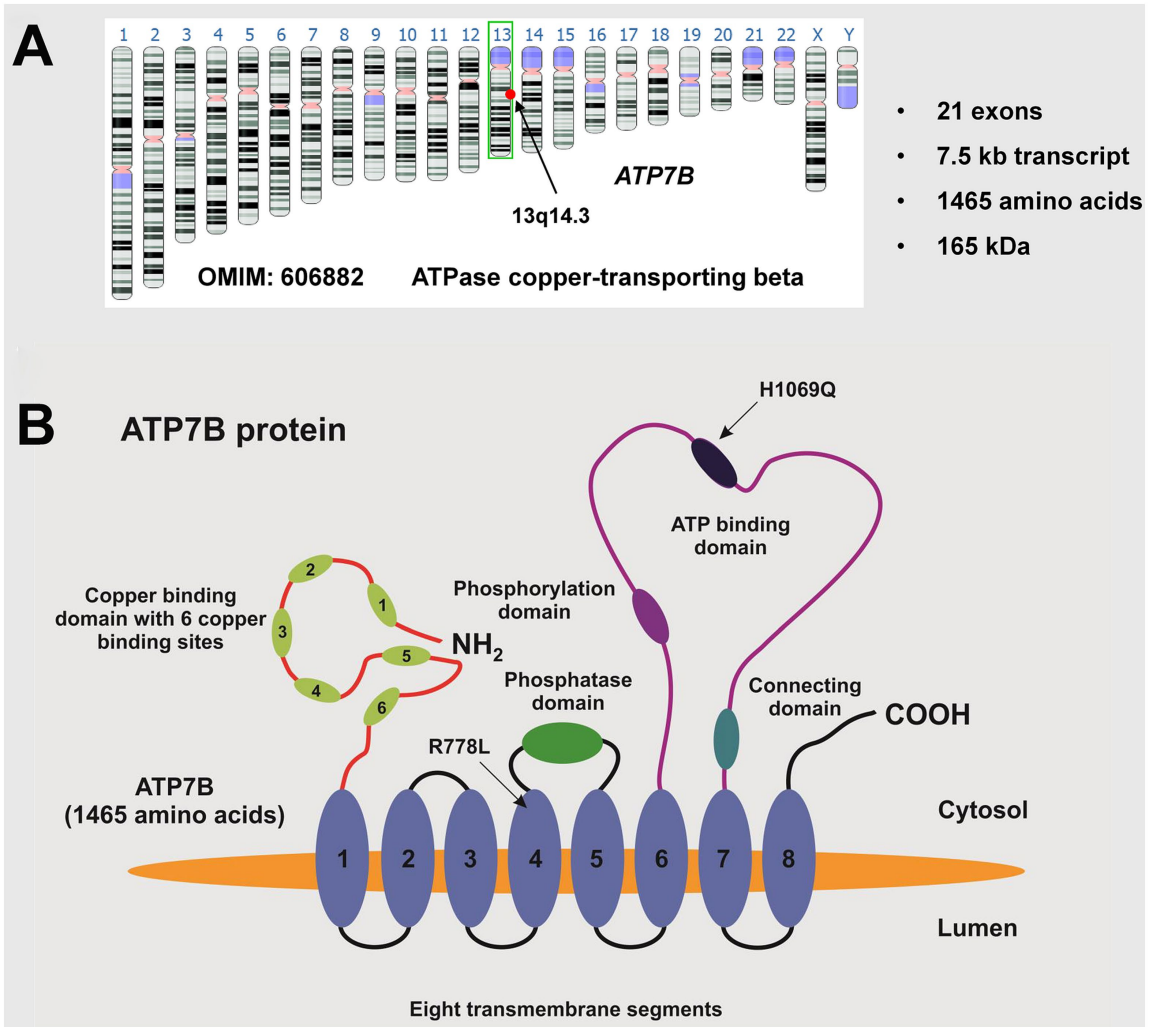
The *ATP7B* gene. **(A)** The *ATP7B* gene (OMIM: 606882) is located on the long arm (q-arm) of human chromosome 13, specifically in region q14.3. It consists of 21 exons, encoding a transcript of approximately 7.5 kb that translates into a 1,465 amino acid protein with an estimated molecular weight of around 165 kDa. **(B)** The ATP7B protein, known as ATPase copper-transporting beta, contains a phosphatase domain, a phosphorylation domain, an ATP binding domain, a metal binding domain, and eight transmembrane segments. The ideogram image shown in **(A)** was obtained from the Genome Data Viewer of the National Library of Medicine (35). There are over 500 known disease-causing mutations, with the most common European mutation being H1069Q and the most common mutation in the Chinese population being R778L. The location of these mutations is shown. This figure was taken from Stremmel and Weiskirchen (9).

The genetic defect in this autosomal recessively inherited disease results from mutations of the *ATP7B* gene (Figure 2). More than 500 disease causing mutations are known in the *ATP7B* gene and no other gene is affected (19–21). It results in the absence or functionally impaired ATP7B, leading to apoceruloplasmin not being appropriately loaded with copper. As a consequence, most of the apoceruloplasmin is degraded (16). The dramatic decrease of ceruloplasmin in blood does not seem to have a significant clinical impact. The decrease of ceruloplasmin lowers the blood copper concentration, reversing the ratio of ceruloplasmin-bound copper to albumin-bound copper from 95:5 under physiological conditions to 5:95 in WD, which is a diagnostic hallmark. Non ceruloplasmin bound copper (NCBC) corresponds to the terms “albumin bound copper” because transcuprein or other sources of loosely bound copper are quantitatively negligible. Many physicians call it “free copper,” which means the same.

The pathophysiology of copper metabolism in WD is illustrated in Figure 1 on the right. Due to the blockade of ATP7B and depleted trans-Golgi network stores, biliary excretion of copper ceases, resulting in copper accumulation in hepatocytes in the pre-Golgi cytosolic compartment. Metallothionein synthesis is stimulated by an excess of cytosolic Cu^+^ and stored therein to manage the copper overload. Additionally, excessive Cu^+^ load is exported from hepatocytes via CTR1 to the blood, increasing free copper (NCBC or albumin-bound copper), which then spreads to extrahepatic organs and triggers a variety of symptoms. The most clinically significant symptom is the involvement of the brain. The basal ganglia are particularly sensitive areas, and damage to them leads to motor disturbances (9, 11). The NCBC also leads to enhanced release in urine, serving as a hallmark for the diagnosis of WD with >100 μg/day. As the liver is unable to properly excrete excess copper through bile, SCC serves as an alternative pathway for the body to eliminate this excess copper via the kidneys into the urine which is a key diagnostic indicator and monitor for the success of therapy (12).

**Figure 3:**
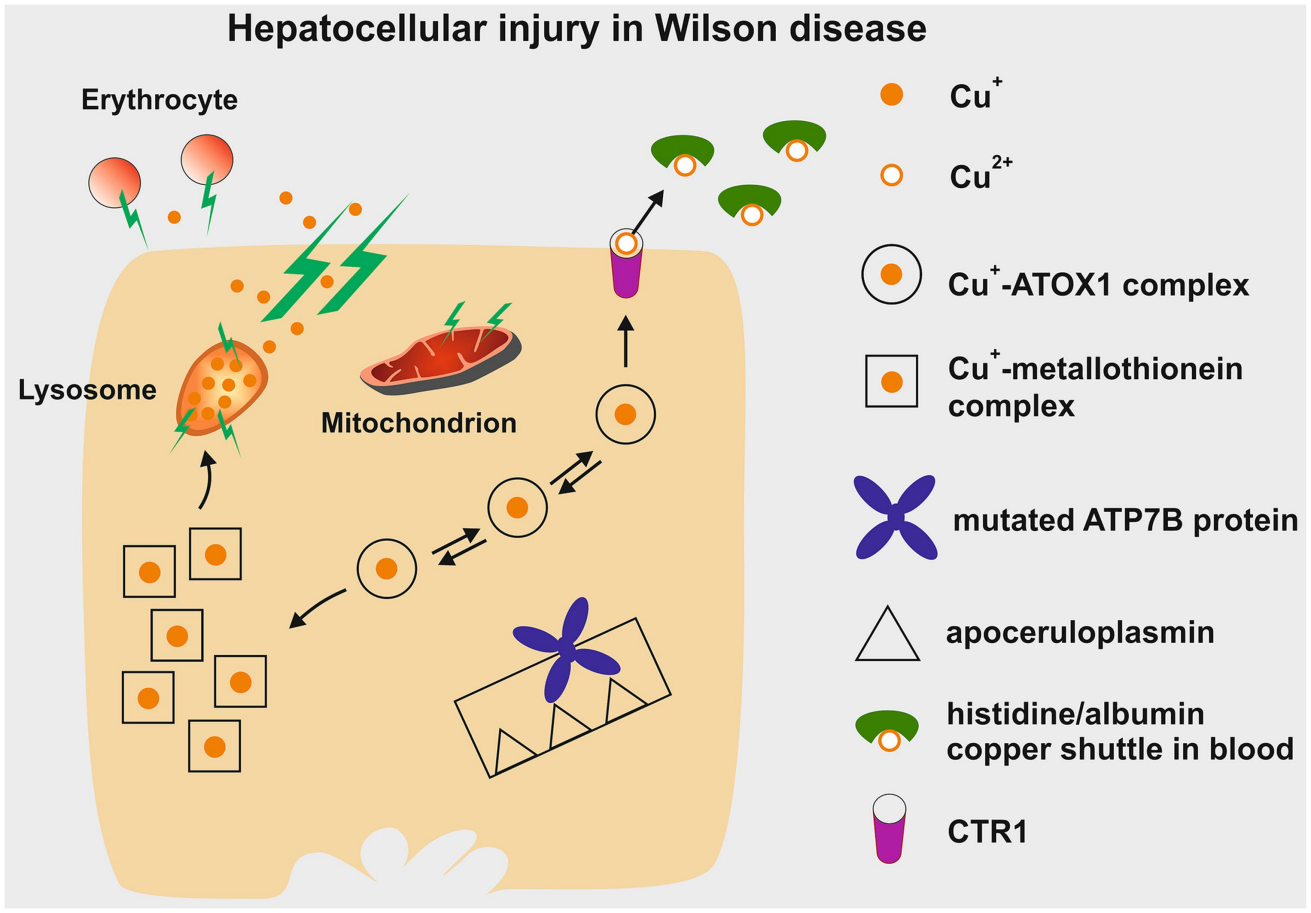
Depiction of the potential hepatotoxic effects of copper accumulation in Wilson disease. Initially, excess copper is sequestered within cytosolic metallothionein. Once these stores are full, copper can be re-excreted through CTR1 into the bloodstream. From there, it is transported as non-ceruloplasmin-bound copper within a histidine/albumin shuttle complex to other organs, such as the brain, and eventually excreted in urine via the small copper carrier. In hepatocytes, additional copper build-up leads to lysosomal deposition, which can be detected by positive Rhodanine staining. The reactive nature of Cu^+^ damages the lysosomal membrane, causing rupture and the release of Cu^+^ into the cytoplasm, resulting in damage to hepatocytes. This process also renders the plasma membrane permeable, allowing Cu^+^ to leak into the bloodstream and contribute to erythrocyte membrane destruction (hemolysis). Cu^+^, cuprous form; Cu^2+^, cupric form; ATOX1, antioxidant copper chaperone; ATP7B, ATPase copper transporter beta; CTR1, copper transporter 1. This figure was taken from Stremmel and Weiskirchen (9).

In the liver, continuous accumulation of Cu^+^ activates increasing levels of protection (Figure 3). When liver cells can no longer handle the excess Cu^+^ through increased excretion via CTR1 and metallothionein synthesis, the Cu^+^ is stored in lysosomes. Copper forms complexes that are positively stained by Rhodanine. Eventually, the toxic Cu^+^ and resulting free radicals cause membrane damage, lysosomal rupture, and the release of lysosomal content into the cytosol. This leads to damage of mitochondria and affects cellular respiration. The plasma membrane then becomes permeable, releasing excessive Cu^+^ into the bloodstream. This can damage erythrocytes, causing hemolysis. The free Cu^+^ can then affect other organs carrying CTR1 (7), with the brain being particularly vulnerable, resulting in severe neurological impairment, especially in the motor system. Urinary copper levels increase significantly as long as kidney function remains intact. Renal failure is rare in WD, but a hepato-renal syndrome may occur in cases of acute liver failure. Normally, copper accumulation in the liver occurs gradually over years, leading to elevated liver enzymes and, due to inflammation, fibrosis and cirrhosis with portal hypertension. Therefore early diagnosis and treatment are crucial.

## 5 Diagnosis

**Figure 4:**
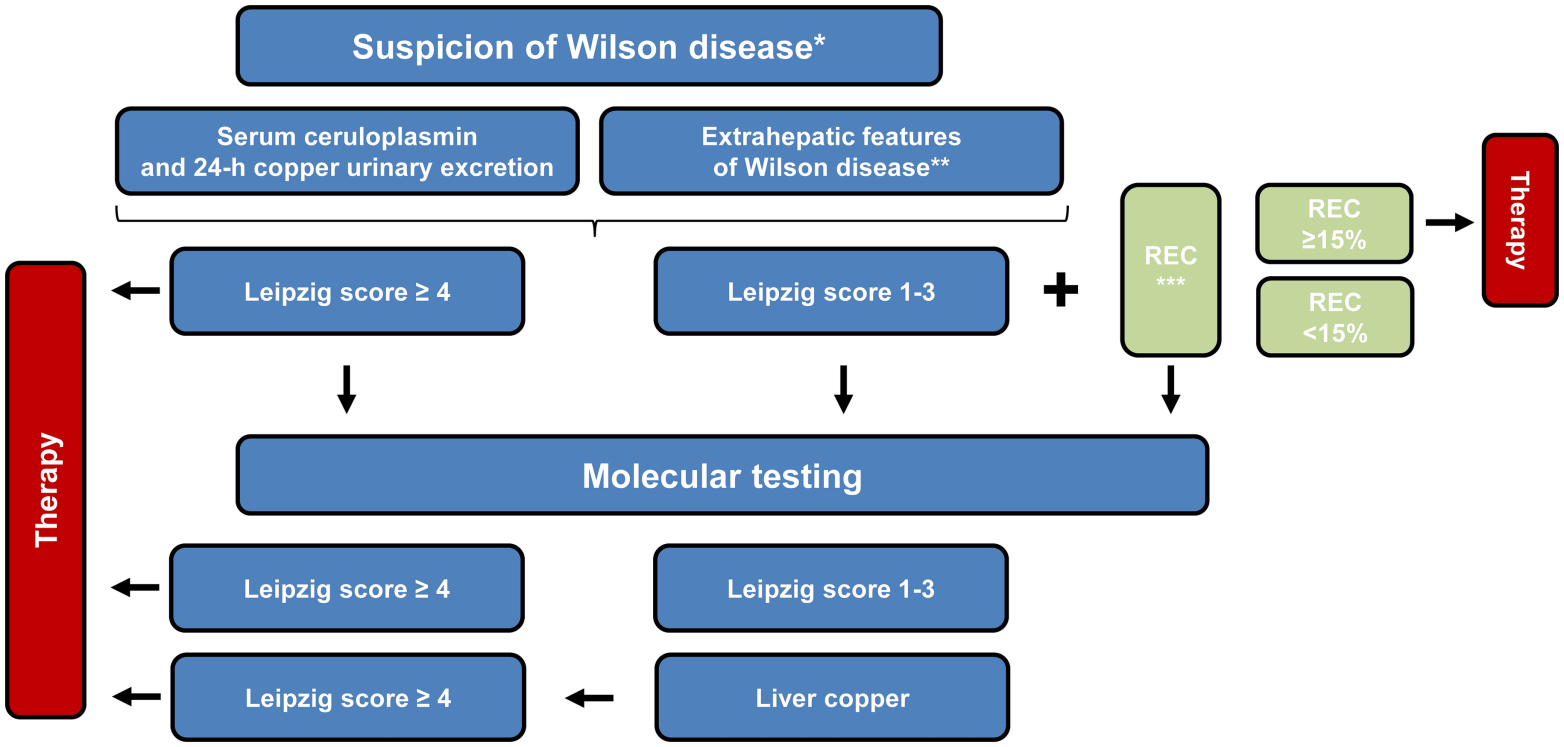
Schematic overview of the proposed diagnostic workflow for Wilson disease. The pathway integrates clinical findings, biochemical parameters and, where appropriate, genetic analyses to guide decision-making. Once an index patient has been confirmed, first-degree relatives can be assessed through tailored approaches, which may involve direct *ATP7B* sequencing, targeted mutation analysis or other genotype-focused strategies. ^*^Diagnosis begins with suspicion and relies on the symptoms that are present. Family screening is crucial in order to consider the medical history of a patient. ^**^Representative clinical “red flags” comprise neurological or psychiatric abnormalities attributable to copper overload, the presence of Kayser–Fleischer corneal rings, and unexplained Coombs-negative hemolytic anemia. ^***^Definition of relative exchangeable copper (REC): the percentage ratio of the exchangeable copper fraction to the total serum copper concentration. This figure was adapted from European Association for the Study of the Liver (5).

Diagnosis of WD can sometimes be a challenge because it is a rare disease and symptoms are subtle. Pure liver involvement is typically without clinical symptoms except for occasional fatigue. Therefore, a routine laboratory examination including transaminases is recommended even in adolescents. The suspicion of WD is high when there is a family history present. Table 1 lists the diagnostic tools and the Leipzig score for establishing the diagnosis (11, 22). According to the pathophysiology, urinary copper excretion and determination of the NCBC are the most sensitive tests for diagnosing WD. Several tests have been developed in recent years to measure non-ceruloplasmin-bound copper for the diagnosis and monitoring of WD. A significant diagnostic advancement was made with the implementation of the direct assay of “free copper,” or exchangeable copper (CuEXC) (23, 24). The methodology for measuring CuEXC through ultrafiltration coupled with atomic absorption spectrometry was first reported in 2009 and applied to healthy individuals to establish reference value ranges (25). The relative exchangeable copper (REC), which is the ratio of CuEXC to total serum copper (Total Cu), has been proposed as a potential diagnostic biomarker for WD (26). REC has been shown to enable a diagnosis of WD with high sensitivity and specificity close to 100% when its value is >18.5% (27). It can differentiate between homozygous, heterozygous *ATP7B* carriers, and individuals with no *ATP7B* mutations, making it useful for family screening. Additionally, individuals with other liver diseases such as metabolic dysfunction-associated steatohepatitis (MASH) or autoimmune hepatitis can be excluded using the critical cut-off value of 15%. The severity of the copper imbalance and the resulting clinical manifestations are reflected in the height of CuEXC. REC can be used as an additional test to the Leipzig scoring system to establish the diagnosis (Table 2 and Figure 4).

**Table 1:** Leipzig scoring system for the diagnosis of Wilson disease^a^.

Typical clinical symptoms/signs		Other tests/score points	
Kayser–Fleischer rings	Score	Liver copper (in the absence of cholestasis)	Score
Present Absent	2 0	5× ULN (>4 μmol/g)	2
		0.8–4 μmol/g	1
		Normal (<0.8 mol/g)	−1
		Rhodanine-positive granules^b^	1
Neurologic symptoms^c^	Score	Urinary copper	Score
Severe Mild Absent	2 1 0	Normal	0
		1–2× ULN	1
		>2× ULN	2
		Normal, but >5× ULN after D-Penicillamine	2
Serum ceruloplasmin	Score	Mutation analysis	Score
Normal (>0.2 g/L)	0	On both chromosomes detected	4
0.1–0.2 g/L	1	On one chromosome detected	1
<0.1 g/L	2	No mutation detected	0
Coombs-negative hemolytic anemia	Score	Total score	Diagnosis
Present Absent	1 0	≥4	Established
		3	Possible, more tests needed
		≤2	Very unlikely

a This information is adapted from European Association for Study of Liver (22).

b If quantitative liver copper levels are not available.

c Or if typical abnormalities are observed on brain magnetic resonance imaging. ULN, upper limit of normal.

**Table 2:** Adverse effects in treatment with D-penicillamine, trientine, and zinc.

Occurrence time	D-Penicillamine	Trientine	Zinc
Early	Fever Diarrhea Rash Nausea	Constipation	Diarrhea Vomiting
Late	Leukopenia Autoimmune diseases Nephrotoxicity Myasthenia gravis Swollen glands Polymyositis Collagen disorders Sensory polyneuropathy	Aplastic and sideroblastic anemia Hair loss Mood changes	Dyspepsia Pancreatic enzyme elevation

In 5% of cases, a fulminant course is observed with clinical deterioration leading to death in the majority of patients, sometimes within only a few days. This acute hepatic failure is described as an apoptotic explosion (22, 28–31). Affected patients are often young between 15 and 20 years old (with a predominance of females) with no signs of liver disease before (32). Kayser–Fleischer rings or motor disturbances are usually absent. As hepatic failure progresses, coma develops. Coombs negative hemolysis with concomitant elevation of bilirubin (conjugated and unconjugated) may occur. Transaminases may only be slightly elevated, and alkaline phosphatase may even be low (28, 29). Coagulation is suppressed, making biopsies for the determination of hepatic copper content a risky procedure. Due to hepatorenal syndrome with anuria reliable data on urinary copper excretion become unavailable. Serum copper levels could be elevated due to the destruction of liver cells and the release of copper. The determination of CuEXC is helpful in this situation. Mutation analysis is often inconclusive and possibly time-consuming. These are the challenges in diagnosing fulminant WD which requires time pressure because only urgent transplantation, requiring a definite diagnosis, can save the lives of the patients (22, 30).

## 6 Treatment options

The concentrations of copper needed for metabolic requirements are low. It is the accumulation of toxic free Cu^+^ that induces cell destruction, starting in the liver. Therefore the goal of therapy is the elimination of this toxic free copper. The development of clinical manifestations typically takes years, usually occurring between the ages of 15–25. In some cases, late manifestations have been reported in individuals over the age of 60, while others remain asymptomatic throughout their lives. This variability is likely due to the type of mutation in the *ATP7B* gene or compensatory mechanisms that are not fully understood.

The most common therapy for WD involves the use of copper chelators, such as D-penicillamine and trientine, which were developed by John Walsh in the 1950s. These chelators promote copper excretion in urine, with D-penicillamine being more effective. This could be due to a lower rate of absorption for trientine. Despite the lower urinary copper excretion capacity of trientine, the therapeutic efficacy is identical. This is due to an inhibitory effect of trientine on intestinal absorption: a dual mode of action not observed with D-penicillamine. Whether this is due to an induction of metallothionein synthesis in the intestine to inhibit copper absorption or the copper chelating effect on intestinally presented copper remains open. The latter mechanism seems less probable because inhibition of absorption was not observed with the stronger chelator D-penicillamine. Both chelators should always be taken with sufficient distance form food consumption (33). The chelator induced increased urinary excretion indicates the presence of free copper in the blood. Continuous use of chelators helps prevent hepatic copper overload and subsequent liver injury. Stopping treatment can lead to deterioration within 18 months and an increased risk of fulminant hepatic failure (22). While chelators are effective, they come with a significant risk of adverse events, particularly in the case of D-penicillamine (Table 2) (34).

Another effective treatment is oral zinc supplementation, which stimulates the production of metallothionein in the intestine and other organs. This helps to inhibit copper absorption and neutralize the toxic accumulation of free Cu^+^ in hepatocytes. However, zinc therapy does not increase copper excretion in urine. Instead, it promotes a negative copper balance by blocking absorption (11, 34). Both zinc and chelators are effective in reducing the toxic free Cu^+^ ions that lead to clinical symptoms. Other treatments, like tetra-thiomolybdate, have shown severe adverse effects, while gene therapy approaches are still undergoing clinical trials. It is important to note that gene therapy may only be administered once due to potential immunologic reactions. The reduction of free Cu^+^ ions remains the optimal treatment choice for WD, a condition that was once fatal and associated with debilitating neurological symptoms before effective treatments became available.

## 7 Take home messages

1. In WD albumin-bound copper, also known as free copper, is increased in the blood and taken up by susceptible organs that carry the copper transporter 1 (CTR1), such as the liver and brain. It also enables some copper release in urine via the Small Copper Carrier.
2. In hepatocytes, the genetic defect in the *ATP7B* gene blocks the uptake of copper into the trans-Golgi network, preventing its biliary excretion and copper loading of apoceruloplasmin. This results in low serum ceruloplasmin levels. The excess cytosolic copper (Cu^+^) is excreted in the blood or stored within metallothionein before being deposited in lysosomes. Excess lysosomal free copper leads to their bursting, causing liver injury and excessive release of free copper in the blood, increasing the risk of hemolysis and copper flooding in other organs.
3. The therapeutic goal is to reduce free copper as early in the course of the disease as possible to prevent manifestation in the liver and brain. Therefore, early diagnosis is the main challenge.

## 8 Remarks

This perspective article summarizes a portion of the webinar titled “*Metabolic Mysteries: The Role of Copper Dysregulation in Liver Disease*,” which was organized by the *Metabolism and Target Organ Damage* on April 17, 2025. The presentations from this webinar can be found at: https://www.oaepublish.com/webinars/mtod.310.

## Footnotes

^1^ https://www.oaepublish.com/webinars/mtod.310
